# Barriers and facilitators to identifying depression in adolescents: A cross-cultural qualitative study in Brazil, Nepal, and Nigeria

**DOI:** 10.1371/journal.pmen.0000209

**Published:** 2024-12-23

**Authors:** Scott A. Collins, Katherine Ottman, Jyoti Bohara, Vibha Neupane, Anna Viduani, Silvia Benetti, Thais Martini, Claudia Buchweitz, Olufisayo Momodu, Abiodun O. Adewuya, Kamal Gautam, Helen L. Fisher, Christian Kieling, Valeria Mondelli, Brandon A. Kohrt, Syed Shabab Wahid

**Affiliations:** 1 University of Colorado School of Medicine, University of Colorado, Aurora, Colorado, United States of America; 2 Center for Global Mental Health Equity, The George Washington University School of Medicine and Health Sciences, Washington, District of Columbia, United States of America; 3 Transcultural Psychosocial Organization Nepal (TPO Nepal), Baluwatar, Kathmandu, Nepal; 4 Department of Psychiatry, Universidade Federal do Rio Grande do Sul, Porto Alegre, Rio Grande do Sul, Brazil; 5 Department of Psychiatry, Lagos Island General Hospital, Lagos, Nigeria; 6 Department of Behavioural Medicine, Lagos State University College of Medicine, Lagos, Nigeria; 7 Institute of Psychiatry, Social, Genetic and Developmental Psychiatry Centre, Psychology and Neuroscience, King’s College London, London, United Kingdom; 8 ESRC Centre for Society and Mental Health, King’s College London, London, United Kingdom; 9 Child and Adolescent Psychiatry Division, Hospital de Clínicas de Porto Alegre, Porto Alegre, Rio Grande do Sul, Brazil; 10 Department of Psychological Medicine, Institute of Psychiatry, Psychology and Neuroscience, King’s College London, London, United Kingdom; 11 National Institute for Health and Care Research Maudsley Biomedical Research Centre, South London and Maudsley NHS Foundation Trust and King’s College London, London, United Kingdom; 12 Department of Global Health, Georgetown University, Washington, District of Columbia, United States of America; CHINTA Research Bangladesh, BANGLADESH

## Abstract

There is growing global interest in early detection and engagement with care for adolescents experiencing depression. However, there is limited information on perceived barriers and facilitators to engagement with adolescent mental health care in low- and middle-income countries. Accordingly, this study examined perceived barriers and facilitators to the identification of depression in adolescents in Brazil, Nepal, and Nigeria. Key informant interviews (n = 153) and focus group discussions (n = 31) were conducted in Brazil, Nepal, and Nigeria with adolescents, parents, health care workers, social workers, teachers, and other stakeholders to explore perceived barriers to identifying depression in adolescents. This study employed a deductive theory-informed codebook enhanced with inductive codes and analyzed using constant comparison by a team of 8 multi-site researchers. The framework approach was used to construct overarching themes and to synthesize a theoretical model. Stigma and a lack of knowledge about the signs and symptoms of depression were perceived as major barriers to the identification of depression across all three settings. Three other themes emerged:(1) role of stakeholders in identifying depression, (2) training or education as a facilitator to identifying depression, and, (3) the role of technology as barrier or facilitator depending on its specific application. Teachers and parents were the primary stakeholders identified as being able to recognize early signs and symptoms of depression in adolescents. Respondents described training through public awareness campaigns, school policy, or social media as interventions to improve depression identification, but noted that social media could also contribute to exacerbating symptoms of depression. These findings suggest there are common perceived barriers and facilitators to the identification of depression in adolescents across diverse cultural contexts. These findings can inform the development of culturally sensitive strategies to address stigma and increase mental health literacy, and ultimately, to improve engagement with treatment and prevention for adolescents experiencing depression.

## Introduction

The prevalence of major depressive disorder (MDD) among adolescents aged 10–19 years old is 19% globally [1]. Additionally, UNICEF’s 2021 report on the State of the World’s children revealed that 166 million adolescents aged 10–19, or 13 percent of all adolescents, across the world were living with a diagnosed mental health condition [2]. Depression and anxiety disorders contributed to over 40 percent of this burden. Given that in lower-resourced parts of the world adolescents are rarely screened for such conditions, the actual figure is likely much higher. Moreover, death by suicide features prominently in the top 5 causes of mortality across adolescence, with one child dying from suicide every 11 minutes. The bleak nature of these statistics is further aggravated when considering that only approximately 0.1 per 100,000 psychiatrists specializing in children and adolescents are available in low- and middle-income countries (LMIC) [2].

Adolescence is a critical period of transition during which young people face significant physical, emotional, and social changes, and are exposed to a range of difficult experiences, such as stigma, bullying, and other life stressors. Unfortunately, the failure to recognize and address depression in this population persists as a global concern [3]. Despite the significant burden of depression among adolescents, many cases go undetected, untreated, or are overlooked due to common misconceptions and misunderstandings about depression specifically, and mental illnesses overall. The provision of evidence-based treatment and care for depression has proven to be difficult thus far, especially in low- and middle-income settings. Depression as it manifests in adolescence is not fully understood, and accumulating evidence suggests that adult models of depression may inadequately reflect adolescents’ experience of the condition [4,5]. Furthermore, the majority of patients seek care later in life, when their depression has advanced, attenuating the effectiveness of treatment options. Most mental health conditions, including depression, manifest before the age of 18 [6]. For example, a robustly conducted prospective longitudinal cohort study identified that most adult mental disorders can be considered as extensions of juvenile disorders that manifested during childhood or adolescence [7]. Accordingly, there have been concerted calls to identify risk factors in adolescents and provide early treatment in order to lessen the strain on health systems and improve quality of life across the lifespan [7,8]. Therefore, it is essential to understand the barriers and facilitators to identifying and addressing depression among adolescents across diverse cultural settings in order to effectively address the global mental health needs of this population.

Despite this call to action, several challenges in accurate identification of depression early in adolescence persist across the globe. Screening tools for depression perform sub-optimally in providing accuracy of diagnosis. In a systematic review of screening tools in the United States, cut-off scores for depression were found to be inconsistent, and a need for exploratory studies was cited to determine more sensitive and specific thresholds [9]. This inaccuracy of existing tools was criticized as potentially leading to overdiagnosis, placing undue burden on health services, or prescription of psychotropic medicine to otherwise healthy adolescents likely increasing exposure to harmful adverse effects. The problem is only compounded when considering the use of such tools in LMIC settings. Cultural heterogeneity of the condition, its symptomatology, actual and perceived etiology, and risk and protective factors, limit accurate detection [10,11]. Treatment effectiveness may also be moderated on the basis of addressing those experiences and patient outcomes held to be salient by global populations [12], and not necessarily only treating symptoms that inform Western disease models.

Cross-cultural research can help identify cultural and contextual influences on depression, including how global populations define, experience, and respond to it. This may be particularly relevant in the field of adolescent mental health, since culture plays a significant role in defining adolescence [13]. However, most psychological research has been limited to high-income countries [14], while 90 percent of the world’s adolescents live in LMICs [15]. Cultural and regional variations in symptom expression, behavioral and psychosocial norms, and illness definitions create significant challenges in identifying and addressing adolescent depression, and therefore often require culture-specific approaches to care [10]. Investigating depression across multiple global settings can provide clearer insight into universal and context-specific risk and protective factors. Moreover, recognizing perceived barriers and facilitators to the identification of adolescent depression in these settings can provide specific targets for future intervention.

In line with this, the goal of this study is to better understand the facilitators and barriers to identifying and treating adolescent depression in diverse cultural settings by using a qualitative approach and involving participants from Brazil, Nepal, and Nigeria. This study aims to gain insight into the cultural nuances surrounding depression identification and treatment engagement to inform the development of culturally compelling interventions based on local experiences and priorities [16,17].

## Materials and methods

### Study settings

The study settings for this research include three diverse cultural contexts: Brazil, Nepal, and Nigeria. These settings were chosen because they represent a range of economic conditions and social settings that are relevant to understanding the perceived barriers and facilitators to the identification of depression in adolescents.

#### Porto Alegre, Brazil

Brazil is an upper-middle-income country, reflective of BRICS (Brazil, Russia, India, China, and South Africa) nations, with a rapidly developing economy and urbanization, and a population of 212.5 million, over one-fifth of whom are between the ages of 10 and 24 [18]. The Brazilian arm of this study was conducted in Porto Alegre, with a population of 1.4 million and a similar proportion of individuals between the ages of 10 and 24 [19]. Public education and health care are guaranteed rights in Brazil, but despite this, a significant portion of adolescents struggle to complete or have dropped out of school [19], and there are significant challenges in the access to specialized mental health services for children and adolescents [20,21].

#### Nepal

At the time of the study Nepal was a low-income country with a diverse population and a complex history. The country is a resourced-limited setting, and the experiences of adolescents living in Nepal reflect the challenges faced by youth in the least-developed nations in a South Asian context. For example, although Nepal is a country of roughly 30 million individuals, it has only one 50 bed mental hospital [22]. Additionally, Nepal has recently emerged from a civil war and frequently faces environmental disasters, making it representative of the experiences of adolescents living in humanitarian settings. Adolescents age 15–24 make up 19% of Nepal’s population [23]. The Nepali arm of this study was conducted in the greater Kathmandu area. Studies have shown that psychosocial problems are prevalent among adolescents in Nepal, with 14% of youth reporting at least one such issue [24] and more than 10% reporting functional difficulty with anxiety or depression [25].

#### Nigeria

Nigeria is a lower-middle-income country and the most populous country in Africa. The population of Nigeria was 194 million at the time of the study. In Nigeria, a significant portion of the population falls within the adolescent age range, with 20% of the total population between 15 and 24, and 42% of the population being under the age of 14 [26]. Adolescents in Nigeria commonly face mental health issues, such as ADHD, neurological conditions, depression, and psychotic disorders [26,27]. However, access to care for these mental illnesses is limited due to a combination of financial, institutional, stigma-related, and sociocultural factors [28,29]. The Nigerian component of this study was conducted in Lagos, the most populous city in Nigeria, and one of the most populous cities in Africa [30]. The city continues to experience rapid population growth due to migration from other parts of Nigeria and other African countries [15,31].

### Study design, sampling, and data collection

This study was designed according to the Framework Method [32] to explore adolescent and adult stakeholder experiences and opinions regarding barriers and facilitators to identifying depression early in adolescents. For adult participants, this study utilized focus group discussions (FGDs), and qualitative interviews with informants including health care workers (e.g. psychologists, social workers, and physicians), teachers, parents, and adolescents. The interview guides are available in an additional file (see S1 Text). Recruitment and data collection for the study was conducted from 30-12-2018 to 31-01-2020.

The data presented in this study form part of the broader research effort by the Identifying Depression Early in Adolescence (IDEA) consortium. The IDEA project is a multi-site research project aimed at pinpointing effective strategies for early identification and treatment of adolescent depression [10,33]. It emphasizes the development and testing of new tools and approaches, alongside providing relevant training and education to healthcare providers and families, to better support young people with mental health concerns. The consortium’s work operates within diverse cultural contexts and includes different research methodologies, facilitating a comprehensive understanding of perceived barriers and facilitators to identifying adolescent depression. A specific focus is placed on understanding adolescent experiences, mental health nuances, and culturally specific risk and protective factors. Also, there is an emphasis on addressing the lack of feasible tools for early depression risk determination before the disorder develops. The outcomes of IDEA studies underscore the crucial role of understanding cultural contexts in developing tools for early identification and prevention, ultimately aiming to improve mental health outcomes for adolescents. The detailed protocol for the qualitative components of the IDEA project are elaborated upon in a separate publication [33].

The participants in this study were chosen based on their personal experience with adolescent depression or their influential role in the lives of adolescents. Adolescents with a history of depression and parents of depressed adolescents were specifically targeted for recruitment using a purposive sampling method. Depressed adolescents were diagnosed with clinical interviews conducted by local mental health clinicians familiar with the local culture. Policy officials at relevant government agencies, including social services, health and education ministries or bureaus, were also purposely selected for participation. Health care workers, teachers, social workers, and policy makers were recruited for this study through convenience sampling, using the researchers’ professional networks and referrals among recruited individuals. Recruitment efforts included both in-person and electronic outreach.

The development of the key informant interviews (KII) and FGD guides was informed by Engel’s biopsychosocial risk factors approach [34] and Kleinman’s explanatory model framework of mental illness [35]. The guides were developed by the research team and reviewed by two senior researchers (CK and BAK; CK is a child and adolescent psychiatrist; BAK is a psychiatrist and medical anthropologist). Six pilot interviews were conducted in each site to test the guides, and they were subsequently adjusted to consider cultural and contextual aspects. The primary areas explored in the KII and FGD were the experiences of adolescents regarding depression and barriers to the identification of depression in adolescents perceived by all participants.

In Brazil, 54 KIIs were conducted with participants including health workers in the public and private sectors (n = 12, 11 females), teachers (n = 12, 10 females), social workers (n = 12, 11 females), policymakers (n = 6, 2 females), parents of adolescents with depression (n = 6, 4 females), and adolescents with lived experience of depression (n = 6, 4 females). Two focus groups were also conducted, one with adolescents (n = 5, 2 females) and another with parents (n = 6, 5 females). Adolescents were between 14 and 17 years of age (mean age = 15.4 years) and all attended public state schools in the city of Porto Alegre. Eight adolescents reported lived experience of depression (all adolescents participating in the KIIs and two from the FGD). All the adolescents included (n = 11) had a mental health-related history of service use, however, clinical diagnosis confirmation was not ascertained prior to their inclusion in the study. All interviews were conducted in Brazilian Portuguese at the Hospital de Clínicas de Porto Alegre or other convenient locations, such as private practice settings. The interviews were conducted by three female researchers (AV, SB, and TM; AV was an undergraduate student in psychology during the study period; SB has a PhD in child and family studies, and TM has a PhD in psychiatry and behavioral sciences) trained in cross-cultural psychiatry research methods. Additionally, the two focus groups, one with adolescents and one with parents, were conducted simultaneously (but in separate rooms) also at the Hospital de Clínicas de Porto Alegre. The focus groups and interviews ranged from 40 to 90 minutes. They were audio-recorded and later transcribed in Brazilian Portuguese for analysis. Field notes from interviews were preserved after each interview/discussion.

In Nepal, a total of 60 KIIs were conducted with adolescents who had depression (n = 7), healthy adolescents (n = 3), and adolescents with depressive symptoms who were referred for evaluation (n = 2), as well as parents (n = 6), teachers (n = 10), social workers (n = 14), primary care providers (n = 6), mental healthcare providers (n = 6), and policymakers (n = 6). Additionally, two FGDs were held with parents (n = 12) of both depressed and non-depressed adolescents. The semi-structured interviews were conducted at various locations in the Kathmandu area, including a non-profit organization focused on psychosocial well-being, partner hospitals, and respondent offices. The interviews were conducted in either Nepali or English, depending on the preference of the respondent, by three female study authors (KO, JB, and VN; KO has a Master of Public Health degree; JB & VN have Bachelor of Science degrees in social sciences) and a male research assistant who are all trained in cross-cultural psychiatry research methods. The duration of the interviews was around 45 minutes on average with interviews recorded and later translated from Nepali to English for analysis. Field notes were documented by the interviewers after each session.

In Nigeria, participants recruited for the study included teachers (n = 13), social workers (n = 12), mental health specialists (n = 9), other health workers (n = 5), policy makers (n = 4), and parents (n = 3). These participants were selected based on their direct experience with or knowledge of adolescent depression or their role in shaping the lives of adolescents. Due to restrictions placed by the Nigeria partner institutions ethical requirements, adolescents could not independently participate without having a caregiver present. Therefore, we did not proceed with adolescent interviews as there was a potential risk of bias asking about mental health in front of their caregivers. Data was collected through KIIs (n = 39) and FGDs (n = 8). The Nigerian component of the study was advertised in key community spaces to recruit parents of adolescents with a history of depression. Despite considerable efforts, we faced difficulty in recruiting parents of children with depression resulting in a lower number of parents included in the study for Nigeria than originally planned. Other participants were chosen by headmasters of government-owned and private schools, and by referral from initial participants. The study included one-on-one interviews with adult stakeholders and one FGD. All interviews were conducted by a Nigerian female psychiatrist, a Nigerian male Psychiatrist and PhD researcher (OM and AA, respectively), and a Nigerian female research assistant, in spaces to promote confidentiality, audio recorded in English and professionally transcribed. Interviews and focus groups lasted approximately 45–90 minutes in duration. Field notes were also documented by the interviewers after each session.

### Data analysis

We used the framework method to guide data analysis [32], which offers a systematic sequence of procedures to analyze qualitative data. Subsumed under the broader set of thematic analysis methods, the framework method does not necessarily prescribe to any particular epistemological ideas, but rather focuses on flexibility and pragmatism that can be used to inform multiple qualitative approaches to develop themes. The prescribed steps of the framework method [32] were systematically applied, namely: (1) transcription, (2) familiarization with the transcripts, (3) coding, (4) developing an analytical framework, (5) applying the analytical framework to the transcripts, (6) charting the data into a matrix, stratified by relevant criteria (for the current study, data was stratified according to respondent type, with a primary focus on adolescent narratives where available, in contrast to adult stakeholders’ perspectives), and (7) interpretation of the data.

The framework method was deemed appropriate for the current study as it has been cited to be especially useful where multiple researchers are engaged in data analysis, involving multi-disciplinary research teams, with varying degrees of expertise or prior experience of qualitative research, to manage data analysis procedures cohesively [32]. The framework method holds utility for managing large datasets, as is the case for the current study, where the goal is to synthesize and derive comprehensive and crosscutting perspectives reflecting areas of convergence while preserving nuanced differences across the entire dataset.

The study used a deductive theory-informed codebook that was modified to include inductive codes and categories. We used seminal theoretical frameworks such as Kleinman’s explanatory model [34] and Engel’s biopsychosocial risk factors [35], to inform a set of deductive a priori codes, and added salient respondent-driven inductive codes to the codebook, as identified during coding. The utility and process of generating theory-driven deductive codes has been described in the literature previously [36], and a deductive-inductive hybridized approach to analysis has been demonstrated to increase rigor in thematic analysis [37].

The use of the framework method has been cited [32] as apt for this sort of hybrid inductive-deductive analysis due to its analytic flexibility, where the research question determines the inclusion of a priori deductive codes derived from previous knowledge and theory, while leaving room for the identification of inductive codes during the coding process.

A team of eight (AV and SB in Brazil; JB and VN in Nepal, and KO and 2 female research assistants in the United States) researchers applied the final codebook to the full dataset using NVivo version 12 [38]. Inter-rater reliability (IRR) of 0.7 (Cohen’s Kappa) was established among the coders, indicating substantial agreement [39]. IRR use has been cited to improve the communicability, transparency, and trustworthiness of qualitative analysis, especially for cross-cultural studies which collect and analyze data in multiple languages and uses multiple coders [40]. A constant comparison approach was used during coding, where newly coded data were compared to previously coded segments to ensure consistency of code usage across the dataset [41]. Inductive codes and themes were introduced until no new codes or themes could be identified, indicating theoretical saturation [42]. Code queries were executed in NVivo and code summaries were written and categorized to capture different stakeholder perspectives, including stratifying code summaries by adolescents and other stakeholders. In Nepal and Nigeria, transcripts were translated into English and analyzed. In Brazil, data was analyzed into Portuguese, and code summaries were translated into English to inform study outputs. The analysis process was managed by SSW, a doctoral candidate in global health at the time of the study period, who is trained in cross-cultural psychiatry qualitative methods. Overall guidance and supervision of the study was provided by senior researchers, BAK (MD, psychiatry; PhD, medical anthropology), CK (MD, psychiatry), HF (PhD, psychological medicine), AA (MD, psychiatry), KG (MD, psychiatry) and VM (MD, psychiatry; PhD, psychological medicine).

For the current study, codes that were most likely to capture the perceived barriers and facilitators to identifying depression were utilized. These included barriers to identification of depression, role of stakeholders, distinguishing depression from other experiences, training for depression identification, and the role of technology in depression screening and care provision. Codes were synthesized by SAC (medical trainee and PhD student) in consultation with BAK and SSW and refined by other authors to produce an overarching theory that connected the identified relationships among various categories and themes, focusing on barriers to identifying adolescent depression, as reported by participants. This study followed the Consolidated Criteria for Reporting Qualitative Research (COREQ) checklist (Tong et al., 2007 [43]) with details in an additional file (See S2 Text).

### Ethics

The institutional review board of the George Washington University provided full ethical approval for all three sites of this study (180417). The study also has ethical approval in Nepal provided by the Nepal Health Research Council (Registry: 395/2018; Approval Reference: 809). Ethical approval in Brazil was obtained from the Hospital de Clínicas de Porto Alegre Ethics Committee (03220818.0.0000.5327). In Nigeria, the Lagos State University Teaching Hospital Research and Ethics Committee, along with The Research and Ethics Committee of The Federal Neuropsychiatry Hospital Yaba, Lagos, provided full ethical approval of this study. All procedures performed in this study followed the ethical standards of these institutions. All researchers engaged in data collection were trained in recognizing mental distress, debriefing skills, and how to refer to care, if necessary. Any participants with mental health concerns for themselves or on behalf of their children were referred to local mental health providers. Prior to the interview, written consent was obtained from respondents. For children (ages 18 and under), oral assent was obtained, and written informed consent was obtained from the child’s parents. To ensure the protection of confidentiality and full anonymization of the data, any identifiable details given by participants during their interviews were omitted in the interview transcripts. Participants were also assigned codes to protect their identities. All study participants were provided information on the study aims and objectives, informed of their right to refuse participation and to end the interview at any time.

## Results

The details of respondents who participated in this study are provided in Table 1. Adolescent and adult respondents across all three countries in this study expressed the lack of knowledge and understanding of depression, and of mental health in general, as primary barriers to identifying depression.

“I wanted people to try to understand the other side, to try to understand what they [depressed people] are going through. If not, there’s no way… Because they don’t know what people are going through. They don’t know what goes on in people’s daily lives.” (Adolescent 9 in Brazil)

“It’s only the people that have the knowledge that will be able to identify [depression]: ‘Oh, this child is suffering from depression.’ So, ignorance can be a major [barrier] because most people don’t know if they can identify depression or not.” (Social Worker 9 in Nigeria)

**Table 1:** Sample characteristics from Brazil, Nepal, and Nigeria (KII & FGDs).

	Brazil n (%)	Nepal n (%)	Nigeria n (%)
Adolescents			
Age (mean)	15.4	18.5	n/a
Gender			
Female	6 (54.5)	10 (83.3)	n/a
Male	5 (45.5)	2 (16.7)	n/a
Adults			
Age (mean)	Not reported	37.8	Not reported
Gender			
Female	39 (81.3)	28 (46.7)	20 (47.6)
Male	9 (18.7)	32 (53.3)	18 (42.9)
Not reported	n/a	n/a	4 (9.5)
Respondent type			
Parents	12	18	3
Educators & School workers	12	10	13
Social workers	12	14	12
Mental healthcare providers	n/a	6	9
Health workers	12	6	5
Policymakers	6	6	4

In stories of successful identification of depression, adolescents often described experiences of stakeholders such as parents or teachers being the first to recognize changes in their behavior. According to these accounts, adult stakeholders can help adolescents to identify their depression and seek professional mental health care.

“I didn’t realize I had depression. My mother saw the changes in my behavior and brought me here for counselling. She was out of the valley for her work and then she went to India for her study for about a year and when she came, she felt that my behavior was changed and then she brought me.” (Adolescent 6 in Nepal)

This central narrative of the relation between awareness and recognition of depression by various stakeholders guided the data analysis of adolescent and adult stakeholder experiences. The descriptions of lack of awareness were common across accounts from Brazil, Nepal, and Nigeria. This generated five main themes related to barriers or facilitators for identifying adolescent depression: (1) awareness of depression; (2) role of stakeholders; (3) stigma; (4) training; and (5) the role of technology.

### Theme 1: Lack of awareness of depression as a barrier to identification

Adolescents and adult stakeholders from all three countries strongly expressed that lack of awareness, knowledge, or understanding of depression was a primary barrier to identifying depression. This lack of awareness was described on the part of all stakeholders, including parents, teachers, friends, community members, and adolescents themselves.

“No, there wasn’t a teacher that came to me and said, “Hey, is there something happening, why are you missing school?” No. The only thing I heard was “Oh, you’re going to fail, your attendance is low. Oh, you never come to class.” That were the only things I heard. I think that if at least one of them paid attention to what was happening, the girl would not have committed suicide.” (Adolescent during FGD in Brazil)

“I didn’t understand my depression. No one helped me understand it, or maybe I didn’t want to understand it. That is why the identification of my depression was delayed. So, people in school should be made aware about depression and the ways to identify it…” (Adolescent 6 in Nepal)

#### Subtheme: Increasing awareness as a facilitator to identifying depression

Adolescent and adult respondents agreed that increasing awareness of the signs and symptoms of depression would help to identify it earlier. By creating an environment in which family members, teachers, and adolescents themselves could feel more comfortable talking about mental illness, respondents predicted that more adolescents would come forward about their depression or that adults would be more likely to express concern on behalf of an adolescent displaying signs of depression.

“Awareness, awareness. We need to continue to create awareness because if you create awareness you demystify it and you have the opportunity of challenging and correcting the myths. You have the opportunity of talking about it no longer in hushed tones but out in the public space. What that does is it empowers people to feel a little bit more comfortable to come out. They know where the locations for help are. So, awareness I think that is one of the huge things.” (Health worker during FGD in Nigeria)

#### Subtheme: Difficulty distinguishing depression from other experiences as a barrier to identifying depression

A majority of respondents indicated that the duration of sad feelings is what distinguishes depression from normal sadness. Not all respondents indicated specific timeframes, but many suggested that lingering sadness that lasts more than 2 weeks could be considered as possible depression. With responses to specific events, such as a loss of a loved one, some respondents suggested a longer, “acceptable” timeframe should be applied, during which sadness can occur without it being considered depression.

“…We have to observe the person for about one month or 2 to 3 weeks at least to know whether or not that person is depressed.” (Adolescent 7 in Nepal)

“Sadness is something that lasts a few days and then you get over it. But when it becomes too long, about 2 weeks, 1 month. Then people will see…changes in you. That is when depression is seen.” (Social worker 1 in Nigeria)

Other Nigerian respondents suggested that regular sadness most often has a short-term, external cause, such as an immediate need or desire being unfulfilled or the loss of something or someone. In contrast, depression was considered more internal in origin. It may have stemmed from sadness from an external event, but several respondents suggested that self-esteem, thought patterns, and how individuals react to their sadness over the course of a longer time contribute to its transition into depression.

“Sadness! We can also say when you’re sad, you know when you’re [grieving a loss] and when you are depressed are very different things…When you are sad, maybe someone did something wrong to you, or maybe you needed something you didn’t get, you feel sad… But when you’re going through depression, the way you think changes, the way you react to situation changes, your affection changes, your mood changes, you know the love for things [you used to love] changes, but it’s different from when you’re sad. When you go through depression, depression on its own is a sickness, it’s an illness.” (Social worker 6 in Nigeria)

Several respondents across all three study sites discussed the difficulty in being able to distinguish potential symptoms of depression from what is considered “normal” adolescent behaviors in each context. They emphasized that the key to assisting with this problem is to understand as much about the adolescents’ individual situations as possible in order to determine whether help is needed.

“The barriers are, sometimes, people not asking [for more details] when you see a more isolated teenager, thinking that, ‘Ah, it’s a teenager thing, right?’” (Teacher 4 in Brazil)

“We do not know if there is a risk or not, whether this is just a teenager thing. I think… adolescent [behavior] is something mixed with symptoms of illness. So, I think that fine distinction is missing, and that is why there is a lack of information about what really discriminates a teenager with his disappointments, with his intensity and such, from what is really a mental health problem.” (Psychologist 11 in Brazil)

“Adolescents are grumpy, that’s their job description, that’s what they are. So we’re looking for that change that’s different, that’s more than what we had before, it’s persistent, [it can include] loss of interest, inability to enjoy activities, there could be decreased energy; all the other [normal adolescent behaviors] could go alongside these relevant symptoms that are known with depression.” (Social Worker 1 in Nigeria)

“Especially over years when we talk about depression mostly the symptoms are very mixed in fact. That’s why the broad questionnaire was developed because if we try to find typical depression cases it is very hard to find because we will find mixture of anxiety, depression, worries—everything. They come up together. It’s very hard to find typical depression.” (Psychologist 8 in Nepal)

This was in part due to lack of knowledge about normal psychological development but also lack of awareness of depressive symptoms and inherent similarities between some aspects of these two processes. Finally, respondents explained that depression itself can be associated with a lack of motivation to seek help on the part of depressed adolescents themselves.

“In a person who is suffering [from depression]…the level of all willpower goes down, and if there is no motivation at all for recovery then without accessing those available services, the situation remains the same.” (Adolescent 3 in Nepal)

Depression was described by Nepali respondents to be different from normal sadness in two ways. While unexpected hurtful life events can bring sadness, it usually goes away in a few days. However, in order to be depression, both Nigerian and Nepali respondents shared that the sadness had to be more severe and had to persist for greater than two or three weeks. Some respondents also included physical symptoms, staying quiet, and irritability, along with the sadness, as depressive symptoms. One respondent in Nepal mentioned that depressed people ‘take tension’ suggesting that the tension idiom has multiple uses, ranging from normal worry and distress to psychopathological affective states. Interestingly, one respondent also mentioned that the actual word ‘depression’ is used in normal conversation to communicate a normal and far less severe distressed states like the distress experienced before an exam, and accordingly, depression and sadness are not always recognized as different things colloquially. ‘Deep’ tension or thinking about a stressor constantly was described by some respondents to lead to depression.

“If one accepts the saying life goes on and feels sad for a while on losing something or missing one’s goal, then that is normal sadness. But when that same sadness… is from inside and exists for a long time, then they determine ‘pervasive and persistent sadness’. These are features of depression.” (Health Worker 14 in Nepal)

“Others might understand ‘tension’ and ‘depression’ similarly, but we see the two terms completely different from one another. Tension is a part of life, which might be positive, negative, short, or long lasting, easy to cope, and people are somehow able to manage their tension. Sometimes, we see tension more frequently in our daily life. However, depression is a kind of disease, which is not manageable at individual level for some people, affects the daily activities and pose risk as other diseases do. Similarly, depression is a kind of mental problem, which needs medication, counseling, or psychotherapy.” (Social Worker 30 in Nepal)

When discussing what distinguishes depression from the normal experience of sadness encountered in life, respondents stated that normal sadness occurs when adolescents’ expectations for certain events, goals, or relationships are not met. This period of sadness can be overcome with minimal effort or subsides within a short period of time. However, when certain adverse events cause intense and persistent sadness which can hamper daily functioning lasting two weeks or more, it is said to be depression. This view was communicated by stakeholders in both Nigeria and Nepal.

Several respondents from Nigeria and Nepal described a lack of screening as a barrier to identifying depression or improved screening as a way to improve identification of depression in adolescents. One respondent from Nigeria suggested regular school screenings as a possible facilitator to improve identification.

The only thing is, if somebody has some symptoms and sometimes, they go to pediatricians or general doctors and if they are aware of it then they may refer to us. We are not screening anyone. (Psychologist 4 in Nepal)

I think there are quite a number of tools available to us to screen you know, not waiting to pick up a problem but to actually screen in at-risk populations. Adolescents are at risk of depression so why wait until you have full blown symptoms? (Social Worker 3 in Nigeria)

### Theme 2: Role of stakeholders in facilitating identification

In both specific stories and broad descriptions of their work and life experience, respondents described groups of important stakeholders for identifying depression in adolescents in Brazil, Nepal, and Nigeria. The stakeholder groups endorsed by the most respondents were parents and families in general, followed by teachers and school counselors.

“I had talked with my teacher as well recently. Some one or two days earlier I talked with my teacher–there was a fight between my mother and father at home. I cried while explaining that and my teacher suggested that I take counseling” (Adolescent 3 in Nepal)

“The good thing about my school is that the teachers pay a lot of attention to each student’s performance… My grades dropped, and there was a teacher who came to talk to me, wanting to know what was happening, and I got everything out of my chest and he helped me to get through what was happening.” (Adolescent during FGD in Brazil)

Most respondents seemed to indicate that parents or teachers were often the first stakeholders to notice behavioral changes in depressed adolescents, and then subsequently referred them to others for care and management. Several mentioned this could be in part because they are the ones who are spending the most time with the adolescents, and therefore will be most likely to notice behavioral changes.

**Subtheme: Noticing behavioral changes as facilitator to identifying depression.** Respondents reported that behavioral change observation by family members, teachers, or friends is a key component of the identification process. Because most adolescents spend the majority of their time between home and school, respondents believe that parents and teachers share the primary responsibility for observing behavioral fluctuation and initial identification. Once there is a reasonable suspicion for a mental health condition, these stakeholders refer to health institutions, hospital, or other mental health service provider (e.g. psychiatrist, psychologist, counsellor, social worker) for formal diagnosis and management.

“…They end up trivializing some behaviors as common components of adolescence, isolating themselves, not wanting to leave, just being on the internet, as normal behavior of this age group. But these may be behaviors that already indicate some preview of a possible depression. (Teacher 3 in Brazil)

#### Subtheme: Depressive symptoms themselves as barriers to recognition

Adults and adolescents across study sites describe the difficulty in recognizing depression due to the similarity between some signs of depression and socially desirable or neutral behaviors. For example, in Nigeria, remaining quiet and withdrawn at school or at home may be seen as good behavior rather than a sign of a problem. And in Brazil, social withdrawal was described as a barrier to noticing behavior change.

“Children that act according to the rules, they are quiet. Now if a child starts becoming depressed, part of the signs and symptoms is withdrawal to oneself. Everybody will be happy: ‘Ah he’s no longer that very noisy child. He is now quiet and he is becoming very responsible.’ We don’t pay attention to the cause. What if there’s something wrong?” (Health Worker 27 in Nigeria)

“I think that most of the time [asking for help] doesn’t happen, because perhaps one of the characteristics of depression is that it immobilizes you, you know, so, depressed young people, they don’t seek help. They lock themselves in, you know, they go out of circulation and, if there is no one monitoring them, very close to them, the risk is that no one will notice.” (School Counselor 5 in Brazil)

### Theme 3: Stigma as a barrier to identification

Respondents considered stigma to be a barrier to identification. Adolescents noted that they are less likely to speak up about their depression if they feel like they will be discriminated against. Respondents across all countries agreed that stigma creates a social environment that makes it difficult for adolescents to discuss their mental illness with other stakeholders.

“…Only after my friend shared their problem with me, we went to the hospital. But people think that if they share their problems with their friends or other people then they might backbite about them and drop some negative comments towards them. This could be because of the fear of sharing…” (Adolescent 7 in Nepal)

“Yeah, because a lot of people suffering on this thing will not want to be stigmatized. That is why most of them will not want to air out their opinion because they…don’t want to be tagged that maybe “I’m sick” or maybe “something is wrong with me and they’re scared”. So, stigmatization is a factor because people don’t want to talk because they don’t want to be stigmatized, they don’t want to be tagged that they have this problem.” (Social worker during FGD in Nigeria)

Adolescents in Brazil agreed that stigma surrounding mental health and depression still exists, even though it is not as common as it was in previous times. To them, Brazilians still have prejudices against mental health topics and professionals, which is still very connected to beliefs that mental health care is only for crazy and delusional. This seems to happen a lot more with older people, but they also recognize a lot of their peers still have misconceptions about mental health and bully adolescents with depression. They also mention social media as one of the most used mediums to talk about mental health—either in the form of online bullying those who have depression but also as a way to share information about mental health and start the conversation.

“I think it’s [stigma towards mental illness] gotten better, but it still exists. I’ve heard many people talking about it on social media, on YouTube about it, but there’s people who see depression as something that’s not important. Leave it, there’s no problem, nothing’s going to happen. But if not enough attention is paid, the person will end up doing things they should not do.” (Adolescent 2 in Brazil)

“I had a neighbor who had just had a baby. And she said ‘I’m not letting my baby bear you, you’re going to hurt him.’” (Adolescent 6 in Brazil)

In Nigeria, many respondents reported that stigma also exists for the family, making it more difficult for parents to play a role in identifying depression among their children. Additionally, some respondents described the role of the clergy in punishing families who have depressed children.

“It’s just something that we don’t talk about. When people are having mental health issues, it’s something that is seen as a shame that the family isn’t handling their business well enough. Even the parents would feel it as a kind of failure reflecting on them. Nobody wants to talk about it because of the churches will capitalize on it and cast and bind.” (Health worker 25 in Nigeria)

### Theme 4: Mental health training

The majority of respondents distinctly mentioned that teachers, health professionals, parents, religious leaders, general members of the community, and adolescents themselves should have some kind of training for mental health awareness which could help with depression identification.

“When there is awareness of the knowledge of depression, it will help to identify depression better. And then when there is training for different levels, we’ll also be able to identify depression as soon as possible… A good teacher should be able to take note of all those things and try to identify what is going on with the pupils, especially when you notice a student is not in a good mood, not responding to questions very well, or you see some other things… When we have training at all levels it will be easier for us to identify depression.” (Social Worker 1 in Nigeria)

Adolescents believe that training for identifying depression should center around increasing awareness about what depression is. There are several elements they would like to see in a depression awareness program. The first is that it is not only physical illness that is important, but also mental illness which needs equal attention. Second, respondents want the public to be educated about the definition of depression and that depression is an illness. Third, they want the public, particularly parents and teachers, to be aware about what the signs and symptoms of depression look like. Finally, several respondents stressed the importance of informing the public about what services are available for those suffering from depression and where they are located.

“First of all, people must have knowledge about depression because people could not identify unless they know about it. People in different places must be made aware about depression and make them know that not only physical health but mental health is also important.” (Adolescent 7 in Nepal)

Additionally, many of the respondents in Nigeria and Nepal focused on the role of training in reducing stigma related to depression. Respondents who mentioned this agreed that conducting some kind of training related to mental health will help in creating awareness, and this ultimately helps in stigma reduction.

“We need to provide training, like awareness training and the training of professionals. Train professionals, train teachers, because these are the areas we can go to cover and to reduce stigma. Even in churches, we need to in a way, train religious leaders.” (Social worker 1 in Nigeria)

Brazilian participants believe information is key and that schools have an important role to play, discussing mental health and stigma and giving adolescents correct information on the topic. They also stress the need for schools to get involved in cases of online bullying, especially those who are directed at people at risk for depression or those who already are.

“I think depression is still a taboo to be discussed. So I think the biggest challenge is being able to overcome this taboo, so that this tool [respondent is referencing a prototype risk calculator for determining risk of developing depression in the future] is actually used, in the best possible way, and that it reaches the adolescents who need to pass this test so that they can be guided to seek help.” (Social worker 12 in Brazil)

"I think talking about the subject is very important, right? I think it’s a subject that needs to be discussed mainly in schools, be it early childhood education or later, in high school. I think it’s a subject that has to be discussed, it has to be addressed constantly. To start breaking down these barriers of stigma. So that people can look at this, see how important it is to stop this behavior. I think that schools, whether children’s or otherwise, have a huge commitment to this. Why not… even before arriving at the psychiatrist, before arriving at the health center, first she will signal her relationships. And we will only be able to see this in education. Or it’s not a family context, right, but if the family already has this very strong, then probably try to be in that school context.” (Social worker 8 in Brazil)

### Theme 5: Role of technology

The role of mobile technology and the internet was positively described by the participants when discussing the possibility of improving education and self-awareness about depression. Self-help groups and social-media platforms were mentioned as tools to improve awareness as well help people with depression.

“This [technology] should be available as central hub where any issues on mental health or queries will be available on one website or one information hub, even if detailed information is not clear there. Information about places where services will be made available must be made clear.” (Adolescent 3 in Nepal)

Another positive use of social media were the personal postings of well-known figures about their own struggle with depression. However, although participants highlighted the positive use of the internet and technologies to improve awareness, education, and detection of depression, they also pointed that the use of social platforms and other media as causes of depression.

“Social media can help… Yeah, they can help, like, bringing information, but they also can do harm. Because people, like I said, there’s a lot of people that show themselves too much, and then the person starts feeling inferior…” (Adolescent 8 in Brazil)

## Discussion

**Fig 1:**
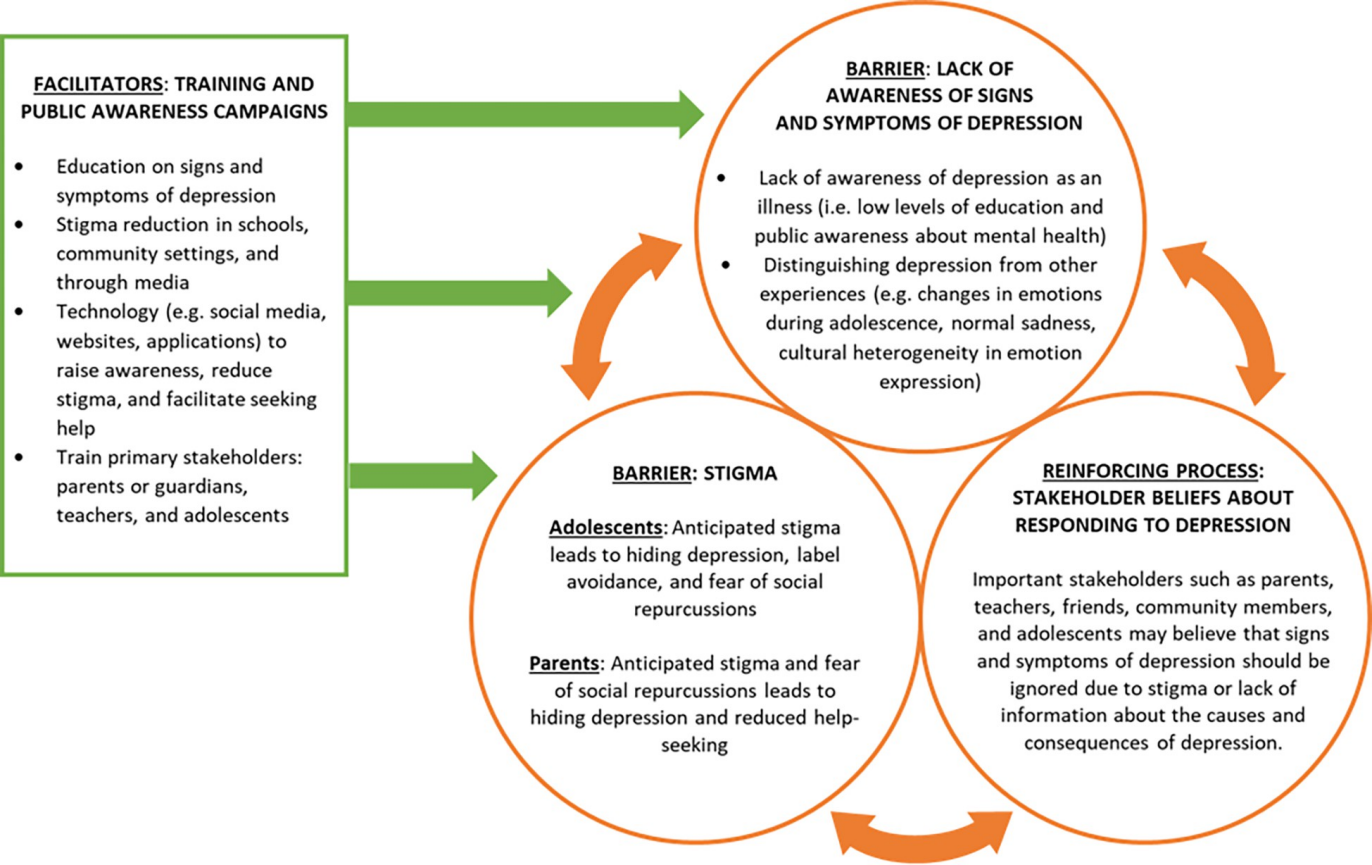
Barriers and facilitators to identifying depression in adolescents in Brazil, Nepal, and Nigeria.

This study provides insights into the perceived barriers and facilitators to identifying depression in adolescents in Brazil, Nepal, and Nigeria. Five overarching themes emerged across all three study sites: (1) awareness of depression; (2) role of stakeholders; (3) stigma; (4) training; and (5) the role of technology. While respondents proposed many potential facilitators that would help identify depression early in adolescents, they revolved mainly around raising public awareness, addressing stigma, and training stakeholders such as parents and teachers about how to identify and seek treatment for depression. Proposed methods for implementing these facilitators included multiple media platforms such as social media, television, and radio to raise awareness regarding signs of adolescent depression and reduce stigma. See Fig 1 for a visual summarization of central themes of barriers and facilitators and their relationships to one another.

When describing their experiences with identifying depression, lack of awareness was a main feature described by both adolescents and adults. Together, these findings highlight the possibility of enduring symptoms as both culturally and clinically relevant, and more generally, a shared sense among respondents of the importance of addressing lack of awareness of signs and symptoms of depression in adolescents. These findings hold several implications for professional practice and public mental health service delivery. As respondents across the sites use various culturally salient terminology for describing depression, primary care providers who may be the first point of contact for such adolescents need to be trained to engage service users in describing their mental and emotional experiences to probe for underlying clinical symptoms. Sharing of idioms of distress, such as ‘tension’ as recounted by adolescents in Nepal, may be indicative of underlying clinical conditions as well. It has been recommended previously that such terms may present a non-stigmatized way to broach the topic of mental health during the clinical encounter in South Asian qualitative literature [44,45]. This may be expanded upon across these cultural settings and tested to see if such an approach leads to better diagnostic accuracy. Similarly, for public mental health interventions, incorporating language and culturally salient terminology used by communities to describe their depression or other mental health conditions has been recommended for cultural adaptation of interventions, as it increases community acceptability [46]. Recently, ‘heart-mind tension’ has been successfully incorporated in a cultural adaptation of a mental health intervention in Nepal [47]. This approach could be used in the other settings of this study to examine if it leads to better acceptability of mental health interventions.

Incorporating improved screening practices can significantly enhance the identification of depression among adolescents in LMICs. While not a primary theme in this study, several respondents proposed that practices such as school-based screenings could offer a secure and less stigmatizing means to identify adolescent depression. This approach is endorsed by findings from the IDEA Delphi consensus study, which highlighted high consensus among global experts regarding the feasibility of school-based strategies and screening tests as detection strategies [48]. Moreover, universal and targeted screening tests improve detection of depression in adolescents and could diminish the negative impact of stigma on help-seeking behavior among adolescents and families [49].

Interventions to detect depression in adolescents in diverse global settings must rely on methodology which incorporates local expressions and conceptions of distress. Locally adapted screening tools or risk calculators could be used in conjunction with the Community Informant Detection Tool (CIDT), an instrument that relies on a vignette and images to describe signs of adolescent depression [50,51]. The CIDT has shown high positive predictive value in detecting adolescent depression in low-resource settings, but is limited as a comprehensive intervention due to difficulty engaging families and adolescents in follow-up, likely due in part to stigma [51]. These interventions could be combined with stigma reduction strategies [52,53], training teachers or spiritual leaders to recognize and respond to depression [27,54], and public awareness campaigns which engage local stakeholders throughout the process to increase cultural acceptability and effectiveness [16,17].

As described in the results of the International Study of Discrimination and Stigma Outcomes (INDIGO), stigma is a common and reversible global barrier to identifying and treating depression [55]. A recent review of stigma-reduction interventions among children and adolescents in LMICs found that all child-focused interventions, including education-based interventions, reported positive results, especially when combined with a face-to-face contact strategy [52]. These results highlight the utility of implementing stigma-reduction programs alongside awareness-raising campaigns and screening interventions, and given the results of this study and of the Delphi study described above [48], these efforts are likely to be successful in a school-based setting.

Technology could play an important role in implementing awareness-raising interventions. Social media, for example, could be used to disseminate information about depression and to raise awareness about the importance of seeking help [48]. Technology can play a key role in implementing public awareness campaigns, contact strategies, and school-based strategies for identifying and addressing adolescent depression. Respondents in the study suggested that utilizing platforms such as social media, television, and radio can be effective in raising awareness of depression and reducing stigma surrounding mental health issues. Additionally, technology can be used to create interactive and engaging educational materials, such as videos and quizzes, that can help increase public understanding of depression and mental health. However, it should be noted that in Nepal, adolescents described social media as a cause of depression due to factors such as social comparison and cyberbullying, which contributed to symptoms of depression through a deep sense of loneliness [56].

The identification, diagnosis, and treatment of depression are parts of a complex process that involves various stakeholders, as highlighted in the results of this paper. Family members, teachers, friends, or adolescents themselves must first recognize the signs and symptoms of depression before considering what next steps to take. However, acting on behalf of an adolescent is not a straightforward decision, as the risk of social repercussions due to stigma must be weighed against the benefits of treatment for both the adult stakeholders and the adolescent. Additionally, the potential benefits of seeking help on behalf of an adolescent must be apparent in order to make the decision to act. To recognize these benefits requires that a stakeholder recognize the danger of depression to the life of the adolescent, understand that depression is a treatable illness, and have access to healthcare providers trained to diagnose and treat depression. Thus, interventions to identify depression in adolescents must target stigma, awareness, and access to resources in order to be successful.

### Limitations

Across all three countries there was a pronounced urban bias, with participants predominantly hailing from urban centers like Kathmandu, Porto Alegre, and Lagos. Such urban-centric samples may not be reflective of rural settings, and therefore, the findings may not accurately capture the diversity of experiences between urban and rural contexts. Additionally, there was a reliance on convenience sampling across the studies, which may have led to results being informed by narratives of respondents who may have been more motivated to participate in the study. In the case of the study from Nepal, there was a higher proportion of female participants, limiting the breadth of perspectives, whereas the Nigerian study solely focused on adult stakeholders, omitting adolescent views due to logistical challenges in conducting research with adolescents in the country.

The term "depression" was broadly used in the Brazilian component of this study without linguistic distinction between feelings of sadness and formal psychiatric diagnosis, possibly leading to culturally-biased perspectives. Additionally, this study had predominant analytical influences from the researchers’ professional backgrounds, emphasizing clinical aspects. This may have overshadowed or misinterpreted culturally-specific signs, symptoms, and experiences of adolescent depression. We acknowledge the need for broader stakeholder involvement in subsequent research, emphasizing a wider contextual understanding of adolescent depression. Lastly, while these studies shed light on local contexts, transferability beyond these specific settings remains a challenge. As such, caution is advised when applying these findings to diverse cultural and socioeconomic settings. Future research should endeavor to address these limitations to provide a more comprehensive and nuanced understanding of adolescent depression across varied settings.

## Conclusion

This study aimed to describe barriers to identifying depression in adolescents in Brazil, Nepal, and Nigeria. Through a qualitative approach, we found five overarching themes that emerged across all three study sites: awareness of depression, role of stakeholders, stigma, training, and the role of technology. These themes highlighted the importance of raising public awareness, addressing stigma, and training stakeholders such as parents and teachers about how to identify and seek treatment for depression. Additionally, the respondents proposed methods for implementing these facilitators, including multiple media platforms such as social media, television, and radio. Based on these findings, future interventions could focus on implementing these facilitators in order to effectively risk-stratify and target preventive interventions for adolescent depression in different cultural contexts. In addition, future studies should be conducted to further explore the effectiveness of these interventions, and to examine specific processes for bridging the gap between identifying risk factors for adolescent depression and referring to specialized care for diagnosis and treatment in low- and middle-income countries.

## Supporting information

S1 Text Qualitative interview guide. (DOCX)

S2 Text COREQ checklist. (DOCX)
